# Case of Glossitis

**Published:** 1849-03

**Authors:** 


### ECLECTIC DEPARTMENT,

### AND SPIRIT OF THE MEDICAL PERIODICAL PRESS.

---

*Case of Glossitis.* By W. Marsden, M. D., Quebec.—Having had two cases of that uncommon, afflicting and dangerous disease, "Glossitis." in the course of my practice, I forward them to you, hoping you will find them sufficiently interesting to give them a place in your journal; and that they may tend to throw some additional light on the pathology of this alarming affection, especially as the results were, in neither of these cases, exactly like any reports that I have yet met with.

Case I.—Miss Lemire, of Grand St. Esprit, æt. 13, called on me at about 11 o'clock, A. M., on Wednesday, the 10th of July, 1844, with a swelled tongue. She was of healthy appearance, full and regular habit of body, sanguine temperament (rather lymphatic), and had not yet menstruated. The history of her case was, that she was walking out on the previous evening in the night air, without bonnet or shawl, and that, just on retiring to rest for the night, she felt slight pain in the tongue, which quickly increased in intensity, and became lancinating; followed by swelling, which also continued to increase during the night, and until the time that she called on me. Her appearance was now peculiarly distressing, and her countenance expressive of the greatest anxiety and pain. Her tongue protruded some distance beyond the teeth, both laterally and at the front; and her breathing was almost entirely carried on through the nose. The teeth were upwards of half an inch apart, and the tongue entirely filled the cavity of the mouth. The tongue presented a remarkable appearance of glossiness, redness, and hardness, and was coated with viscid mucus.

My reading being fresh on this subject, I immediately suspected the nature of the case, although I had never seen one before, and resolved on the mode of action at once; which was effected in less time than I can describe it, and was followed by the most happy results.

Having got the person who accompanied her to secure her head, I opened her mouth as wide as possible, by depressing the lower jaw, and introduced a scalpel along the upper part of the tongue upon its flat surface, and then turning it upon its edge, I cut my way out, by making a deep, full incision into its most elevated part—completely from its base to within less than half an inch of its apex. This incision was immediately followed by a considerable discharge of albumen or coagulable lymph of about the color and consistence of calf's foot jelly, together with about an equal quantity of blood. Her breathing at once became easy and natural, and the tongue returned within its natural and ordinary bounds. She spoke, almost immediately, with surprising distinctness, expressing her grateful acknowledgements for the speedy and effectual relief she had obtained; and she replied, in answer to my interrogatory, that the pain of the operation was comparatively trifling. After washing the mouth for some time with warm water, to encourage the bleeding, if possible, she returned home without adopting any other remedial means, and I did not see her again until the following Sunday, four days after, when the only trace of her sufferings that remained, was a slight mark along the surface of the tongue, a little to the right of the raphe, looking like another raphe, but not the slightest trace of swelling remained. I will now report the other case and reserve my further remarks until I have done so.

Case 2.—Miss M———, ætatis 18, daughter of R. M———, Esq., of the Commissariat Department, was attacked with swelling of the tongue, on Saturday evening, the 9th of July, 1848, having ridden in a covered carriage, the day previously, with the blinds and sides open, and her bonnet and shawl off, to enjoy the air, the weather being warm. This young lady was under the treatment of the medical officers of the army medical staff, from Saturday the 15th of July; and a few days after, also of a civil practitioner, until the 26th of the month, when I was first requested to see her, but declined, for reasons of a private nature with reference to the gentleman last referred to. On the 28th, however, on again being urgently requested to visit her, I did so, at first alone, and afterwards met Dr. M———, D'y. Inspector of Hospitals, who, in common with the other parties that had seen her, entertained the most unfavorable opinion of the case. I will now give her own history of it. She stated that she had, at times, for about five years past, been troubled with swelling of the tongue, which had always subsided previously to the last attack, by the *immediate* application of heat and heated flannels to the head, which were continued for a few hours; but that the right side of the tongue had remained slightly and permanently enlarged since the first attack. She always attributed these swellings to cold; and on the last occasion she cannot account for the obstinacy of the attack.

Among the local applications that had been used by her medical advisers, previous to my being called in, were—blisters to the neck and throat; leeches to the neck, throat, and tongue; warm or hot water injected into the ears, and hot oil; frequent applications of lunar caustic to the anterior and lateral portions of the tongue, which was covered with sloughs; repeated scarification of the dorsum of the tongue with the lancet, poultices, &c. And among the general means employed, croton oil was, I believe, the principal. At the stage of the disease when I first saw her, there existed great nervous prostration, emaciation, and irritative fever, with a quick, small pulse, ranging from 108 to 130.

On the 29th, I again met Dr. M——, and after stating to him the result of my own experience in Lemire's case, besides referring to some published cases, I urged the propriety of one or more free incisions into the substance of the tongue. He replied, "that *he had cut deep enough* with the lancet, if the tongue contained matter;—that it was cartilaginous;—and that it gave no indication of the presence of matter," &c.

I then referred especially to that portion of the case of "Charles Martin, Surgeon of the Island of Mull, reported in the 28th volume of the Edinburgh Medical and Surgical Journal, at page 76, by Dr. John Aitkin," where he says, "previous to the first incision being made, I could not possibly determine, either from existing symptoms, or by external examination, whether or not there was a collection of pus. Could I have satisfied myself of this, I would not have prolonged the patient's sufferings by employing the lancet, but would have had immediate recourse to the knife. As the matter was deeply lodged in the substance of the tongue, I remained ignorant of its formation until the scalpel was used."

I failed, however, to convince him, and the young lady continued suffering until noon the next day, when he had recourse again to small incisions *with the lancet*. At seven in the evening I met him again (although not by appointment) in company with Dr. L., an army surgeon who happened to be on a visit to Quebec, when I again urged my views of the case, in much the same terms I had before used to Dr. M.; but the only result was a repetition of the assertion, that the tongue was cartilaginous—that nothing that had been done for her had been of any service, and that the tongue *did not contain matter*, in which both gentlemen concurred; and on my persisting in urging a free section of the tongue, Dr. M. declared *sotto voce*, "any one may cut deeper that chooses, but I have cut as deep as I dare."

As Dr. M.'s view of the case was hopeless, and yet he would not himself adopt the plan I had suggested, and which I felt confident was the only course that could afford relief, I at once resolved (as the patient was willing) to bear the *onus* of my treatment alone; and almost an hour after this I proceeded to operate.

Having removed the patient from her bed, and seated her in a chair, her father securing the head, I proceeded, as in the former case, to depress the lower jaw, and with some difficulty, (the lancing at noon having increased the swelling)* introduced the scalpel over the dorsum of the tongue, on its

* By making slight incisions or scratches, the inflammatory action and the patient's sufferings are increased—extending the jaws still more apart when they are already painfully wide asunder.

flat surface, and then, turning the sharp edge towards the most elevated part, cut my way out, making an incision, so full, that I could completely bury my index finger in the cavity.* The incision was followed by a discharge of dark black greenish *fœtid matter*, to the extent of about an ounce and a half, (the smell of which impregnated the whole room, so as to render ventilation necessary,) and a small quantity of venous blood.

Therelief afforded was almost immediate, and the expressions of grateful satisfaction and delight of the patient unbounded. She stated that the operation had given her less pain than "the lancings," and that the relief was immediate; whereas, *each application of the lancet was followed by increased pain and swelling*. Among the observations she made, as soon as she could articulate, was this remarkable expression, "That until now it seemed as if the doctors had only been instruments of torture in the hands of the Almighty;" although she neither repined or reproached them, but was thankful for all their unavailing efforts in her behalf, attributing her prolonged and grievous sufferings to some demerit of her own. Subsequently to the operation, I used an astringent gargle, for a few days; and have regularly used the potassæ hydriodas: which she still continues. The tongue is now reduced to its ordinary size and condition; and there is a slight depression about half an inch to the right of the centre of the tongue in the course of the incision made.

Remarks.—There can be no doubt, from the history and treatment of these two cases, that the disease was one of a violently inflammatory character. For a more full history of this disease, I would advise the reader to refer to the case of Martin, before alluded to; and to the *Journal Universel*, &c., June 1823, xxx. 367, which is referred to in the *Edinburgh Medical and Surgical Journal*, page 235, vol. xxi. The author of the paper from which the notice is extracted, refers the reader, in particular, to a treatise on the subject in the 5th volume of the *Memoires de l'Academie de Chirurgie* by de Lamalle, who professes to have derived it from Job-a-Meekren, a well known Dutch surgeon of the 17th century. By this it will be seen that the practice of incisions is not new, but that it was in use at least two centuries ago.

That the inflammation was violent in its character, and rapid in its progress, will appear from all the cases I have referred to. In the case of Lemire, not more than thirteen or fourteen hours after the attack, albumen was largely deposited in the substance of the tongue, and had I not promptly resorted to the use of the knife, the probability is, that in a few hours more the deposit would have been purulent. In Martin's case, the incisions were made about 24 hours after the attack, and the deposit was pus. It will be remarked that the secretion differed in its character in all these cases. In Lemire's the deposit had not yet become purulent, and in Miss M.'s (which was opened three weeks after the first attack) the probability is, that absorption had taken place in some slight degree, which supposition would account for the peculiar character of the deposit, and for the irritative fever under which she laboured.

It will also be remarked, that in each of the reported cases referred to

* In using the word cavity it may be supposed that there was a distinct cavity or cyst in the substance of the tongue: such, however, was not the case, the matter being contained in the cellular substance.

above, several incisions were made, and that I made only one. My reason for so doing was, that in Martin's case, it is stated, that, "I made other two incisions, one in the middle and the other in the left side of the tongue, but no purulent matter followed." Wherefore, I concluded, that there was only one continuous cavity, and that a repetition of the incision would only be attended with useless pain.

It has occurred to me that the disease might be confined to one side of the tongue only, as in both my cases I made my incision in the most elevated part and apparent centre of the tongue, and yet, on the subsidence of swelling, the incision proved to have been on one side. In all cases I have met with, the swelling appears to have been of the right side! Can physiology in any satisfactory manner account for this?

The practice of incisions in swelling of the tongue is neither new nor unfrequent; but the comparative rarity of the disease usually takes the surgeon so completely by surprise, that much time is lost and much suffering entailed upon the patient by delaying its use. I believe it to be the only course that will afford *immediate and permanent* relief.

I will close this lengthy article with an extract from Martin's case, which is so pertinent, that I prefer endorsing it to writing anew my own views. He says, "It is hardly necessary to remind the reader, that to effect any real good, the incisions should be made deep and extensive, otherwise he is only torturing his patient with pain—*likely to prove more injurious than beneficial.** The operation is exceedingly simple, and is not attended with the smallest danger, so that the surgeon *may safely act with boldness and promptitude.* Indeed, such is the urgency of the case, that it imperatively demands vigorous interference; and *trifling remedies are only dangerous instruments* in the hands of a surgeon. In the case above related, it is questionable if the man could have survived for any length of time, unless the enlargement had been subdued by the timely use of the knife; for the *pus was so deeply seated* that he might have been suffocated before it could make its way outwards."

I will only add, that this latter termination is one *we may look for in vain,* as in Miss M.'s case, there was no such attempt, at the end of three whole weeks.—*British American Journal.*

QUEBEC, Sept. 13, 1843.

